# Artificial Diels–Alderase based on the transmembrane protein FhuA

**DOI:** 10.3762/bjoc.12.124

**Published:** 2016-06-24

**Authors:** Hassan Osseili, Daniel F Sauer, Klaus Beckerle, Marcus Arlt, Tomoki Himiyama, Tino Polen, Akira Onoda, Ulrich Schwaneberg, Takashi Hayashi, Jun Okuda

**Affiliations:** 1 Institute of Inorganic Chemistry, RWTH Aachen University, Landoltweg 1, 52056 Aachen, Germany; 2 Institute of Biotechnology, RWTH Aachen University, Worringer Weg 1, 52056 Aachen, Germany; 3 Department of Applied Chemistry, Graduate School of Engineering, Osaka University, 2-1 Yamadaoka, Suita 565-0871, Japan; 4 Institute of Bio- and Geosciences IBG-1: Biotechnology, Forschungszentrum Jülich GmbH, 52425 Jülich, Germany

**Keywords:** artificial Diels-Alderase, biohybrid catalysis, copper enyzme, membrane protein

## Abstract

Copper(I) and copper(II) complexes were covalently linked to an engineered variant of the transmembrane protein *Ferric hydroxamate uptake protein component A* (FhuA ΔCVF^tev^). Copper(I) was incorporated using an *N*-heterocyclic carbene (NHC) ligand equipped with a maleimide group on the side arm at the imidazole nitrogen. Copper(II) was attached by coordination to a terpyridyl ligand. The spacer length was varied in the back of the ligand framework. These biohybrid catalysts were shown to be active in the Diels–Alder reaction of a chalcone derivative with cyclopentadiene to preferentially give the *endo* product.

## Introduction

So-called artificial metalloenzymes have attracted attention over the last decade [1–9]. Incorporation of an organometallic cofactor into proteins offers new possibilities to expand the reaction repertoire catalyzed by natural enzymes to non-natural reactions. With this approach man-made metalloproteins as asymmetric transfer hydrogenases [10–11], Suzukiases [12], metatheases [13–20], epoxidases [21], Diels–Alderases [22–27] and others have been reported. The Diels–Alder reaction is a powerful C–C bond formation reaction, widely used in organic chemistry, e.g., for the synthesis of natural products [28]. This reaction is known to be catalyzed by Lewis acids such as a Cu(II) complex [29]. Additionally, structurally defined catalysts are found to influence the *endo*/*exo* ratio as well as the enantioselectivity [30]. Artificial Diels–Alderases have also been reported to show good *endo*/*exo* selectivities as well as high enantioselectivities in a benchmark reaction of azachalcone with cyclopentadiene [22–27].

The artificial Diels–Alderases reported so far used soluble proteins, where the binding site of Cu(II) was formed either by site-directed mutagenesis [22–23], by incorporation of a suitable ligand, or copper complex in an apo-protein [24–27]. Here we report on the use of the robust transmembrane protein *Ferric hydroxamate uptake protein component A* (FhuA) as host for defined Cu(I) NHC or Cu(II) terpyridyl complexes with a maleimide moiety. By covalently bonding these copper complexes to the protein artificial Diels–Alderases based on a membrane protein have been obtained.

## Results and Discussion

### Synthesis of the metal complexes

**Scheme 1:**
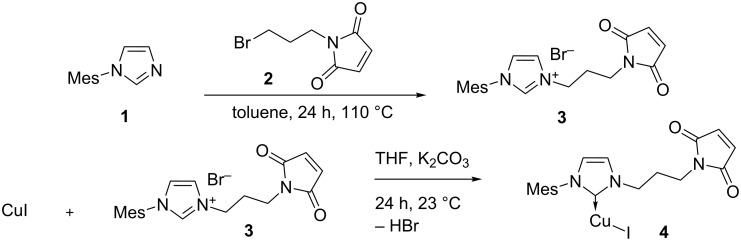
Syntheses to Cu(I) complex bearing a NHC ligand.

As the protein host, the FhuA ΔCVF^tev^ variant of the *Ferric hydroxamate uptake protein component A* (FhuA) was chosen [31]. This protein was shown to be suitable to harbor Grubbs–Hoveyda type catalysts for olefin metathesis [17–18]. To anchor Cu(I) in the protein FhuA ΔCVF^tev^ that contains a cysteine residue at position 545 for conjugation [31], an NHC ligand containing a maleimide function was prepared (Scheme 1).

**Scheme 2:**
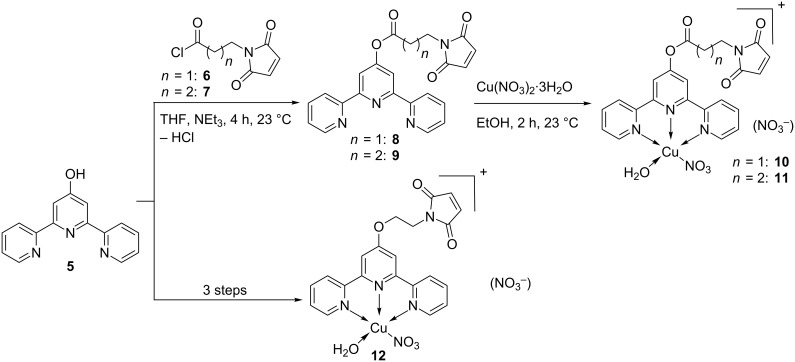
Synthesis of Cu(II) terpyridyl complexes.

The imidazolium salt **3** was synthesized by nucleophilic substitution of mesityl imidazol **1** with maleimide derivative **2**. These salts were used to generate the Cu(I) NHC complexes **4** upon deprotonation with K_2_CO_3_. Complex **4** contains only one NHC ligand at the copper, as shown by elemental analysis and ESIMS. Attempts to coordinate Cu(II) to the NHC ligand failed. However, the terpyridyl (terpy) ligand is a promising candidate to support Cu(II) ions. Therefore, the terpy framework containing an alcohol function on the 4 position of the central pyridine was chosen (Scheme 2).

By either esterification or nucleophilic attack, the spacer with the maleimide group was attached. The ligand was treated with one equivalent of Cu(NO_3_)_2_·3H_2_O leading to the Cu(II) complexes **10–12**.

**Scheme 3:**
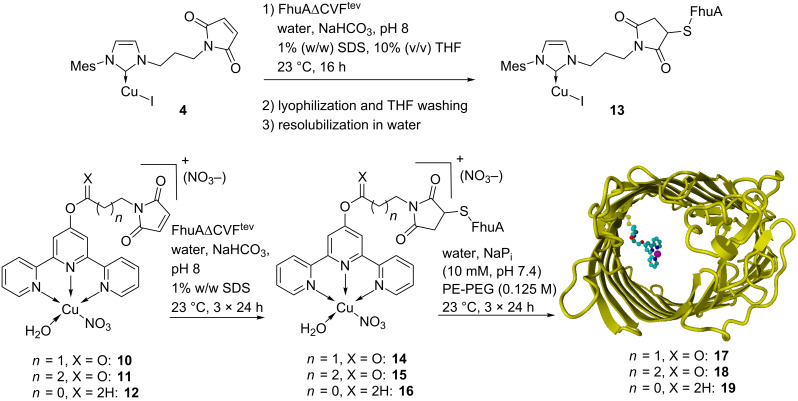
Anchoring and refolding of the biohybrid copper complexes.

By using the established anchoring strategy, the Cu(I) and Cu(II) complexes (**4** and **10–12**) were anchored covalently inside the β-barrel structure. After anchoring, the protein was refolded by dialysis (Scheme 3).

Anchoring of all complexes was successful. Titration of the free cysteine with the fluorescence dye ThioGlo^®^ indicated that more than 95% of the cysteine residues were conjugated for each catalyst.

**Figure 1:**
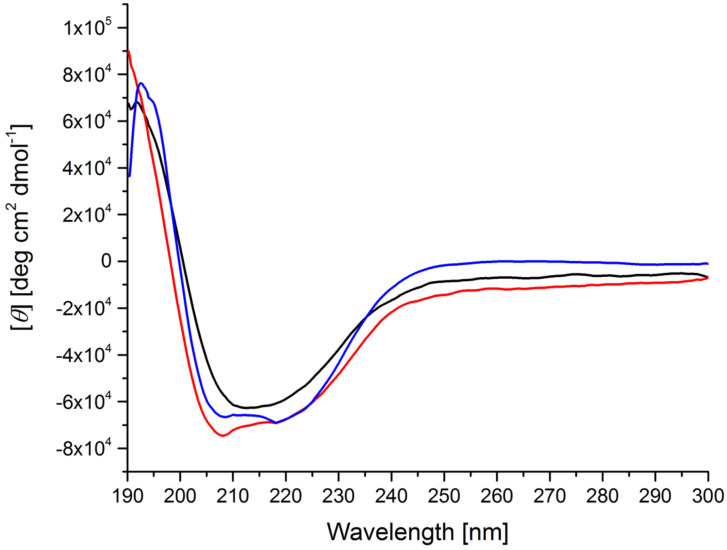
CD spectra of refolded catalysts **17**–**19** (red: **17**, black: **18**, blue: **19**).

Renaturing of the protein was successful in the case of the terpy ligand framework (for clarity of the location of the catalyst, see Figure S1 in Supporting Information File 1). After 3 days of dialysis against SDS-solution, excess catalyst **10**–**12** was removed. Additional 3 days of dialysis against PE-PEG solution renatured the protein structure to give the expected β-barrel structure, as indicated by CD spectra (Figure 1).

The CD spectra show a minimum at around 215 nm and a maximum at 195 nm, as expected for β-barrel proteins such as FhuA [17–18,31]. This finding suggests correct refolding of the protein. Additionally, the temperature stability of the new conjugate **17** was evaluated.

**Figure 2:**
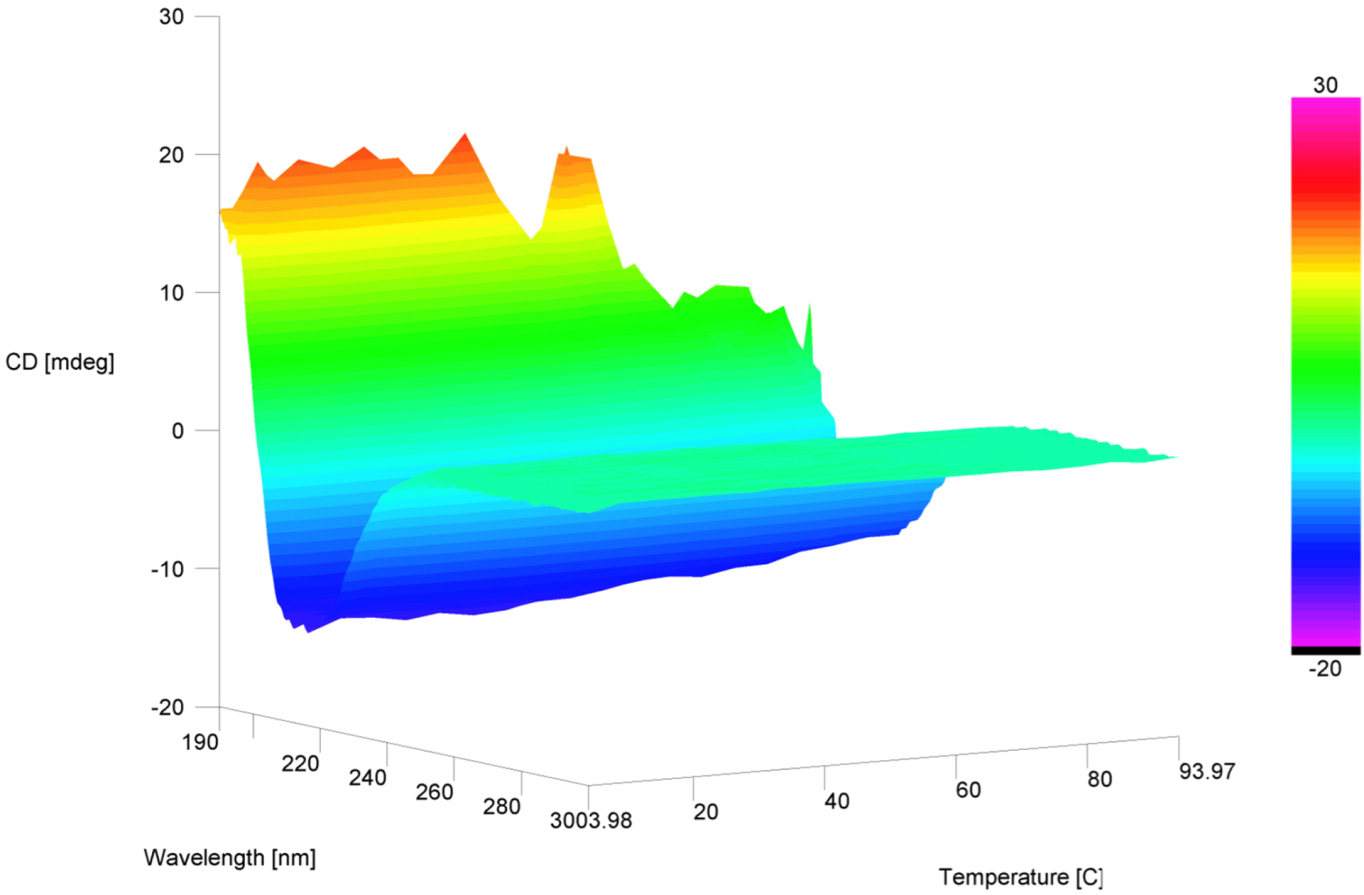
Temperature-dependent CD spectra of catalyst **17**.

The temperature-dependent CD spectra indicate correct folding of the catalyst in the temperature range from 4 °C to 64 °C (Figure 2). This is in agreement with previously reported stability analysis of the wild-type FhuA and the FhuA mutant with its “cork” domain removed (FhuA Δ1-159) [31].

The Cu(I) NHC-containing protein could not be renatured. We speculate that during the refolding procedure Cu(I) was oxidized to Cu(II) by contamination with air. Cu(II) led to protein aggregation and precipitation. This was shown in an independent experiment. When one equiv of Cu(NO_3_)_2_·3H_2_O was added to a solution of FhuA ΔCVF^tev^, the protein precipitated rapidly and quantitatively.

**Figure 3:**
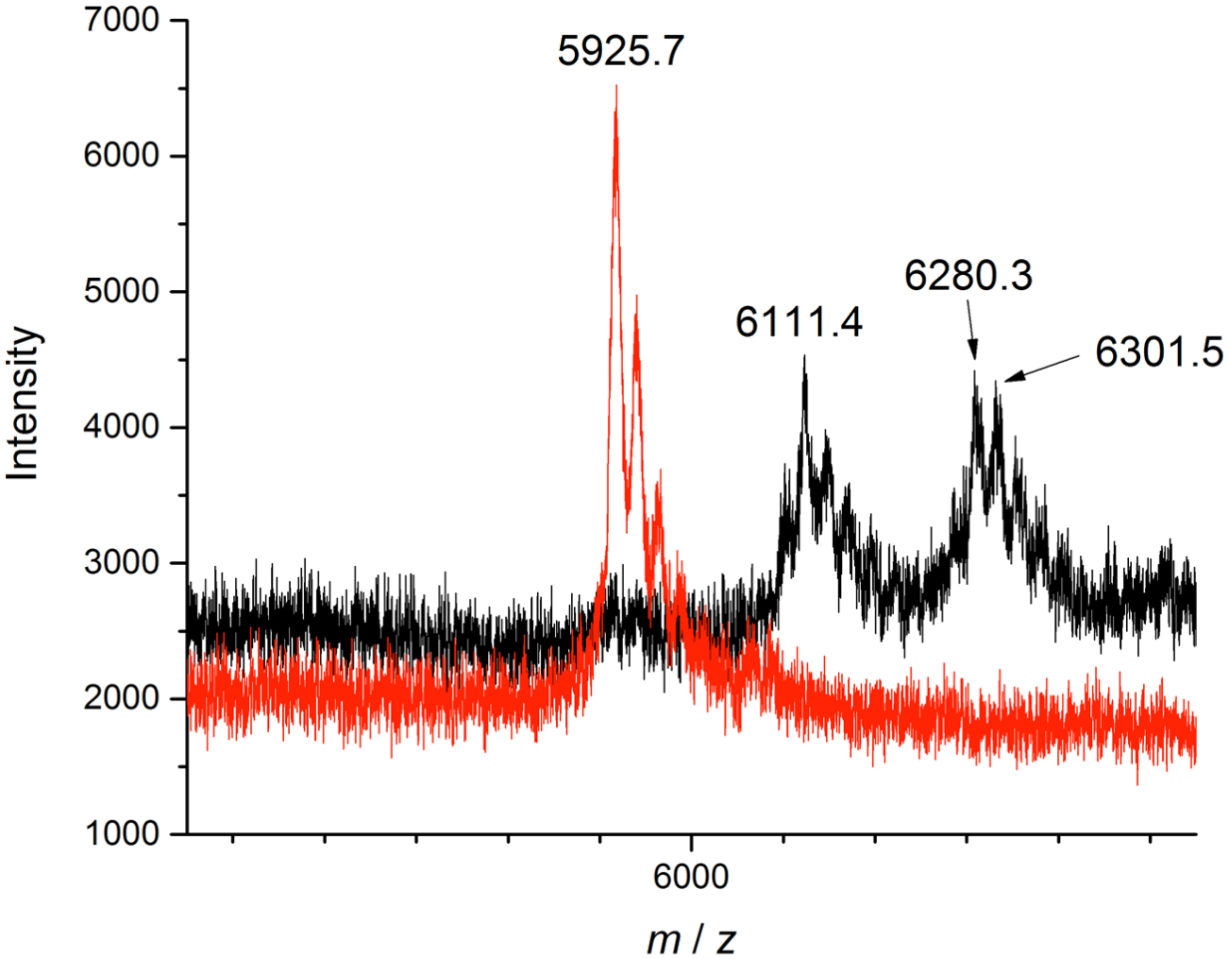
MALDI–TOF mass spectra (black: **17**, red: FhuAΔCVF^tev^).

MALDI–TOF–MS analysis for the whole biohybrid catalyst was difficult due to the high mass of approximately 64 kDa. However, digestion into smaller fragments is possible with the deliberately introduced TEV cleavage site [17–18]. The fragment containing the Cu complex was cut out and analyzed separately (Figure 3).

Digestion of biohybrid catalyst **17** was successful. Comparison of the MALDI–TOF– MS spectrum with FhuA ΔCVF^tev^ (calcd (M + Na^+^): *m*/*z* = 5925 Da; found: *m*/*z* = 5925 Da) indicates successful coupling. The signal of *m*/*z* = 6301 Da indicates the FhuA fragment with the attached ligand framework (calcd (M): *m*/*z* = 6302 Da; found: *m*/*z* = 6301 Da). The signal of *m*/*z* = 6111 Da results from saponification of the ester and the maleimide moiety (calcd (M + H_2_O + Na^+^): *m*/*z* = 6111 Da, found: *m*/*z* = 6111 Da). We were unable to detect the copper ion in the MALDI–TOF–MS.

The isolated biohybrid catalysts were tested in the Diels–Alder reaction of azachalcone **20** and cyclopentadiene (**21**, Table 1). We evaluated first the background reaction of this Diels–Alder reaction in the detergents and buffer solutions we used for the biohybrid catalysts. Since SDS precipitates at 4 °C and the solution becomes heterogeneous, we decided to perform the reactions in SDS at 23 °C. After 3 days, the reaction showed 62% conversion with an *endo* to *exo* ratio of 70:30 (Table 1, entry 2). Since detergents such as SDS influences the reaction significantly, this value is in good agreement with previously reported results [32]. In PE-PEG at 4 °C the conversion was lower; the *endo*/*exo* ratio was ca. 55:45 (Table 1, entry 1). Using CuI in SDS, the conversion increased slightly, showing the same selectivity (Table 1, entry 5). When using Cu(II) as a catalyst, the conversion was complete in both detergent solutions, but no change in selectivity was observed (Table 1, entries 3 and 4). CuI NHC complex **4** showed the same activity and selectivity as CuI (Table 1, entry 6). By using the bioconjugate **13**, the conversion with 62% is comparable with the protein-free catalysts or CuI itself, but the selectivity significantly changed the *endo* product preferred (Table 1, entry 7). By using the Cu(II) complexes **10–12** in the refolding buffer: the conversion decreased with an *endo*/*exo* ratio of approximately 60/40 (Table 1, entry 8–10). Upon attaching the catalyst to the protein in the partially folded state, the selectivity increased to 90% *endo* with high conversions independent of the spacer length (Table 1, entry 11–13). The refolded biohybrid catalysts **17** and **19** showed good conversion with almost quantitative *endo* product formation (Table 1, entries 14 and 16). Catalyst **18** with the longest spacer unit, however, showed moderate activity and loss of *endo* selectivity. This is explained by the high flexibility of this catalyst within the β-barrel structure of the refolded protein (Table 1, entry 15). Based on these catalysis results, we hypothesize that the protein environment is sterically rather demanding, which is even more pronounced in the refolded state. The absence of any enantioselectivity suggests that no preferential orientation of the substrate at the active site within the barrel structure is possible. Notably, no protein precipitated during catalysis, showing the advantageous feature of membrane proteins in terms of robustness as compared to soluble proteins [15].

**Table 1:** Diels–Alder reaction catalyzed by the biohybrid catalysts.

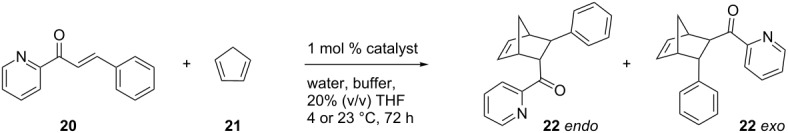					

Entry	Catalyst	Buffer	Temp. [°C]	Conv.^a^ [%]	*endo*/*exo*^b^

1	–	PE-PEG^c^	4	20	55/45
2	–	SDS^d^	23	62	70/30
3	Cu(NO_3_)_2_·3H_2_O	PE-PEG^c^	4	95	54/46
4	Cu(NO_3_)_2_·3H_2_O	SDS^d^	23	94	65/35
5	CuI	SDS^d^	23	78	70/30
6	**4**	SDS^d^	23	75	67/33
7	**13**	SDS^d^	23	62	90/10
8	**10**	PE-PEG^c^	4	21	65/35
9	**11**	PE-PEG^c^	4	33	56/44
10	**12**	PE-PEG^c^	4	12	66/34
11	**14**	SDS^d^	23	92	90/10
12	**15**	SDS^d^	23	87	89/11
13	**16**	SDS^d^	23	91	89/11
14	**17**	PE-PEG^c^	4	69	96/4
15	**18**	PE-PEG^c^	4	15	66/34
16	**19**	PE-PEG^c^	4	64	98/2

^a^Determined by ^1^H NMR in CDCl_3_ and HPLC. ^b^Determined by HPLC. ^c^PE-PEG (0.125 M), sodium phosphate buffer (100 mM, pH 7.4). ^d^SDS (1% w/w), pH 7.5 (adjusted with NaHCO_3_).

## Conclusion

Herein, we report the synthesis of Cu(I) NHC and Cu(II) terpyridyl complexes equipped with a maleimide moiety which underwent covalent conjugation at the cysteine residue 545 of the transmembrane protein FhuAΔCVF^tev^. These biohybrid conjugates were analyzed by CD spectroscopy, MALDI–TOF–MS, ThioGlo fluorescence titration, and BCA assay. All employed methods indicate the folded structure of FhuA ΔCVF^tev^ and a high occupancy of the only accessible cysteine residue within this β-barrel protein.

The biohybrid catalysts showed high activity and high *endo* selectivity in the Diels–Alder reaction of substrate **20** with cyclopentadiene (**21**). A comparison with other reported artificial Diels–Alderases is not meaningful because of the utilization of detergents in the present case, which increases the stability towards the Diels–Alder reaction conditions. However, similar trends with respect to both activity and *endo* selectivity were observed. The cavity of FhuA appears to enhance the reaction as reported by Hayashi et al. for nitrobindin [26], Reetz et al. for serum albumin [22–23], and Roelfes et al. for Lactococcal multidrug resistance Regulator (LmrR) [25]. Furthermore, the increased *endo* selectivity is in agreement with other protein-modified catalysts reported so far [22–27].

## Experimental

### General considerations

All manipulations were performed under argon atmosphere using standard Schlenk or glove box techniques. Prior to use, glassware was dried overnight at 130 °C and solvents were dried, distilled and degassed using standard methods. Catalysis with Cu(II) complexes were performed under ambient conditions. NMR measurements were performed on a Bruker Avance II 400 or a Bruker Avance III HD 400 spectrometer at ambient temperature unless otherwise mentioned. The chemical shifts (δ ppm) in the ^1^H and ^13^C NMR spectra were referenced to the residual proton signals of the deuterated solvents and reported relative to tetramethylsilane [33]. Abbreviations for NMR spectra: s (singlet), d (doublet), t (triplet), quint (quintet), m (multiplet). Elemental analyses were performed on an elementar vario EL machine. CD spectra were recorded on a JASCO J-1100 equipped with a single position Peltier cell holder. MALDI–TOF spectra were recorded on an Ultraflex III TOF/TOF mass spectrometer (Bruker Daltonics). High resolution ESI–TOF–MS were performed on a Thermo Finnigan LCQ Deca XP Plus spectrometer. CuI and Cu(NO_3_)_2_·3H_2_O were purchased from Sigma-Aldrich and used as recieved. Cyclopentadiene was freshly distilled before used. Compounds **1** [34], **2** [35], **5** [36], **6** [37], **7** [37], **12** [26], **20** [32] and FhuAΔCVF^tev^ [17] were synthesized according to literature procedures.

### Syntheses

#### Synthesis and characterization of IMesBr **3**

A solution of 1-(3-bromopropyl)-1*H*-pyrrol-2,5-dione (1.69 g, 7.75 mmol, 1.00 equiv) and 1-(mesityl)-1*H*-imidazole (1.66 g, 8.91 mmol, 1.10 equiv) in toluene (35 mL) was stirred in a closed Schlenk tube for 24 h at 110 °C. The colorless precipitate was filtered, washed with toluene (3 × 15 mL) and dried under vacuum to afford analytically pure imidazolium salt **1** (2.58 g, 6.40 mmol, 83%) as colorless powder. ^1^H NMR (400 MHz, CD_2_Cl_2_) δ 10.32 (s, 1H, NC*H*N), 8.07 (s, 1H, C*H*=CH), 7.28 (s, 1H, CH=C*H*), 7.05 (s, 2H, aryl C*H*), 6.73 (s, 2H, C*H*=C*H*), 4.68 (t, ^3^*J*_HH_ = 6.85 Hz, 2H, C*H_2_*), 3.58 (t, ^3^*J*_HH_ = 6.42 Hz, 2H, C*H_2_*), 2.35 (s, 3H, *p*-C*H*_3_), 2.34 (quint, ^3^*J*_HH_ = 6.72 Hz, 2H, C*H*_2_), 2.10 (s, 6H, *o*-*Me*); ^13^C NMR (100 MHz, CD_2_Cl_2_) δ 171.5 (*C*=O), 141.9, 139.0 (N*C*HN), 135.0 (*C*H=*C*H), 134.9, 131.3, 130.2, 123.8 (*C*H=*C*H), 123.7, 48.0 (*C*H_2_), 34.5 (*C*H_2_), 30.2 (*C*H_2_), 21.4 (*p*-*Me*), 18.0 (*o*-*Me*); ESIMS (+) *m*/*z* (%): calcd for (C_19_H_22_N_3_O_2_)^+^, 324.171; found, 324.170 (100).

#### Synthesis and characterization of NHC-Cu(I)I complex **4**

The Imidazolium salt **3** (200 mg, 0.495 mmol, 1.00 equiv), K_2_CO_3_ (280 mg, 2.02 mmol, 4.00 equiv) and CuI (95 mg, 0.495 mmol, 1.00 equiv) was stirred in THF (5 mL) for 24 h at 23 °C. The solvent was evaporated under vacuum and the residue was dissolved in dichloromethane (4 mL). After filtering over Celite^®^ the solvent was evaporated under vacuum and the residue dried under vacuum to afford CuI NHC complex **6** (150 mg, 0.292 mmol, 59%) as orange powder. ^1^H NMR (400 MHz, CDCl_3_) δ 7.19 (s, 1H, C*H*=CH), 7.28 (s, 1H, CH=C*H*), 6.93 (s, 2H, aryl C*H*), 6.83 (s, 2H, C*H*=C*H*), 4.21 (t, ^3^*J*_HH_ = 6.72 Hz, 2H, C*H_2_*), 3.58 (t, ^3^*J*_HH_ = 6.72 Hz, 2H, C*H_2_*), 2.31 (s, 3H, *p*-*Me*), 2.16 (quint, ^3^*J*_HH_ = 6.72 Hz, 2H, C*H*_2_), 2.00 (s, 6H, *o*-*Me*); ^13^C NMR (100 MHz, CDCl_3_,) δ 181.7 (N*C*N), 170.6 (*C*=O), 139.0, 135.4, 134.9, 134.2, 129.1 121.7, 120.6, 48.3 (*C*H_2_), 34.7 (*C*H_2_), 30.5 (*C*H_2_), 21.0 (*p*-*Me*), 17.9 (*o*-*Me*); Anal. calcd for C_19_H_21_CuIN_3_O_2,_ C, 44.41; H, 4.36; N, 8.18; found: C, 44.02; H, 4.01; N, 7.75; ESIMS (+) *m*/*z* (%): calcd for (C_19_H_21_CuN_3_O_2_)^+^ 388.092; found, 388.106 (100).

#### Synthesis of terpyridyl ligands **8** and **9**

A solution of terpyridine **5** (200 mg, 0.803 mmol, 1.00 equiv) in THF (10 mL) was treated with acid chloride **6** (165 mg, 0.884 mmol, 1.10 equiv) or **7** (177 mg, 0.884 mmol, 1.10 equiv) in THF (5 mL). Triethylamine (NEt_3_) (222 µL, 1.61 mmol, 2.00 equiv) was added to the solution and the mixture was stirred for 16 h at 23 °C. The solution was filtered and all volatiles evaporated. The residue was dissolved in dichloromethane (50 mL), washed twice with water (50 mL), and once with brine (50 mL). The organic layer was dried over Na_2_SO_4_ and the solvent removed under vacuum affording the terpyridine ligand **8** (285 mg, 0.715 mmol, 89%) or **9** (280 mg, 0.699 mmol, 87%). ^1^H NMR (**8**, 400 MHz, CD_2_Cl_2_) δ 8.69 (m, 2H, aryl C*H*), 8.60 (dt, ^3^*J*_HH_ = 8.0 Hz, ^3^*J*_HH_ = 1.0 Hz, 2H, aryl C*H*), 8.26 (s, 2H, aryl C*H*), 7.86 (m, 2H, aryl C*H*), 7.34 (ddd, ^3^*J*_HH_ = 7.4 Hz, ^3^*J*_HH_ = 4.8 Hz, ^3^*J*_HH_ = 1.1 Hz, 2H, aryl C*H*), 6.75 (s, 2H, *H*C=C*H*), 4.00 (t, ^3^*J*_HH_ = 7.0 Hz, 2H, C*H*_2_), 2.99 (t, ^3^*J*_HH_ = 7.0 Hz, 2H, C*H*_2_) ppm; ^1^H NMR (**9**, 400 MHz, CD_2_Cl_2_) δ 8.69 (m, 2H, aryl C*H*), 8.61 (dt, ^3^*J*_HH_ = 7.8 Hz, ^3^*J*_HH_ = 1.1 Hz, 2H, aryl C*H*), 8.25 (s, 2H, aryl C*H*), 7.85 (m, 2H, aryl C*H*), 7.34 (ddd, ^3^*J*_HH_ = 7.4 Hz, ^3^*J*_HH_ = 4.9 Hz, ^3^*J*_HH_ = 1.0 Hz, 2H, aryl C*H*), 6.75 (s, 2H, *H*C=C*H*), 3.71 (t, ^3^*J*_HH_ = 6.8 Hz, 2H, C*H*_2_), 2.67 (t, ^3^*J*_HH_ = 7.4 Hz, 2H, C*H*_2_), 2.09 (pent, ^3^*J*_HH_ = 7.2 Hz, 2H, C*H*_2_) ppm.

#### Synthesis of Cu(II)-terpyridine complexes **10** and **11**

To a solution of terpyridine ligand **8** (200 mg, 0.500 mmol, 1.00 equiv) or **9** (207 mg, 0.500 mmol, 1.00 equiv) in ethanol (10 mL), Cu(NO_3_)_2_**·**3H_2_O (120 mg, 0.500 mmol, 1.00 equiv) in ethanol was added. The solution was stirred for 2 h at 23 °C. The blue precipitate was collected and washed generously with cold THF (20 mL), cold ethanol (50 mL) and cold dichloromethane (10 mL). The residue was dried under vacuum, to give the copper complex **10** (214 mg, 0.365 mmol, 73%) or **11** (207 mg, 0.345 mmol, 69%). ESIMS (**10**) (+) *m*/*z* (%): calcd for (C_22_H_16_CuN_4_O_4_)^+^, 463.0468; found, 463.0459 (35); calcd for: (C_15_H_10_CuN_3_O)^+^, 311.0125; found, 311.0118 (43); ESIMS (**11**) (+) *m*/*z* (%): calcd for (C_23_H_18_CuN_4_O_4_)^+^, 477.0624; found, 477.0624 (7); calcd for (C_15_H_10_CuN_3_O)^+^; 311.0125; found: 311.0114 (36).

### General procedure: Conjugation of the catalysts to FhuAΔCVF^tev^ and refolding

To a degassed solution of FhuAΔCVF^tev^ in water (5 mg/mL, pH ≈ 8 (NaHCO_3_)) containing 1% (w/w) SDS, 10 equiv of catalyst **4** in degassed THF (10% (v/v)) or 10 equiv of catalyst **10**, **11**, **12** in water (10% (v/v)) was added. The solution was allowed to stir 16 h.

In the case of catalyst **4**, water was removed in vacuum, and the residue was washed with degassed THF (4 × 15 mL) to remove excess of catalyst **4**. The residue was dried in vacuum and dissolved in water.

In the case of catalyst **10–12**, the solution was transferred into a dialysis tube and the solution was dialyzed for 3 days against 200 fold volume containing SDS (1% (w/w)) and water (pH ≈ 8 (NaHCO_3_)). The dialysis solution was changed every 12 hours. Afterwards, the sample was dialyzed for 2 days against 200 fold volume containing the refolding detergent PE-PEG (0.125 mM, average *M*_n_ = 2250 g/mol), sodium phosphate buffer (10 mM, pH 7.4), and water. The dialysis solution was changed every 12 h.

The protein concentration was analyzed by BCA assay, the coupling efficiency was determined by ThioGlo fluorescence titration, and correct refolding was determined by CD spectroscopy, as previously reported [17–18]. Digestion of the proteins was performed as previously reported [17].

### General procedure: Diels–Alder reaction

To the corresponding catalyst (1 mol %) in 2 mL of buffer solution (0.125 mM PE-PEG, sodium phosphate buffer (100 mM, pH 7.4), 1 mM EDTA) at 4 °C or 23 °C azachalcone **20** (4 mg, 0.02 mmol) in THF (10% (v/v)) and freshly distilled cyclopentadiene (40 µL, 50 µM, 33 equiv) was added subsequently. The reaction mixture was stirred for 72 h. Afterwards, the mixture was extracted with Et_2_O (3 × 10 mL), the combined organic phases were dried over Na_2_SO_4_ and the solvent removed under reduced pressure. The residue was analyzed by ^1^H NMR spectroscopy and chiral phase HPLC using heptane/isopropanol (98:2) as eluents. All reactions were carried out in triplicates.

### Abbreviations

PE-PEG (polyethylene-polyethylene glycol), SDS (sodium dodecyl sulfate), TEV *(Tobacco Etch Virus),* MALDI–TOF–MS (matrix-assisted laser desorption/ionisation and time-of-flight mass spectrometry), FhuAΔCVF^tev^ (FhuA Δ1-159_C545_V548_F501_tev), CD (circular dichroism), ESI-MS (electrospray ionization-mass spectrometry), NaP_i_ (sodium phosphate buffer).

## Supporting Information

File 1 Illustration of the catalyst **2** and NMR spectra of synthesized compounds.
