# Supplementary material for: Artificial Diels–Alderase based on the transmembrane protein FhuA
Source: Beilstein J Org Chem. 2016 Jun 24;12:1314–21. doi: 10.3762/bjoc.12.124 (PMC4979952; doi:10.3762/bjoc.12.124)
Supplement: File 1 — Illustration of the catalyst 2 and NMR spectra of synthesized compounds. [file Beilstein_J_Org_Chem-12-1314-s001.pdf]

Supporting Information

for

Artificial Diels–Alderase based on the transmembrane protein FhuA

Hassan Osseili‡,1, Daniel F. Sauer‡,1, Klaus Beckerle[1], Marcus Arlt[2], Tomoki Himiyama[3], Tino Polen[4], Akira Onoda[3], Ulrich Schwaneberg[2], Takashi Hayashi[3] and Jun Okuda*,1

Address: [1]Institute of Inorganic Chemistry, RWTH Aachen University, Landoltweg 1, 52056 Aachen, Germany, [2]Institute of Biotechnology, RWTH Aachen University, Worringer Weg 1, 52056 Aachen, Germany, [3]Department of Applied Chemistry, Graduate School of Engineering, Osaka University, 2-1 Yamadaoka, Suita 565-0871, Japan and [4]Institute of Bio- and Geosciences IBG-1: Biotechnology, Forschungszentrum Jülich GmbH, 52425 Jülich, Germany.

Email: Jun Okuda - jun.okuda@ac.rwth-aachen.de

*Corresponding author

‡Equal contributors

Illustration of the catalyst 2 and NMR spectra of synthesized compounds

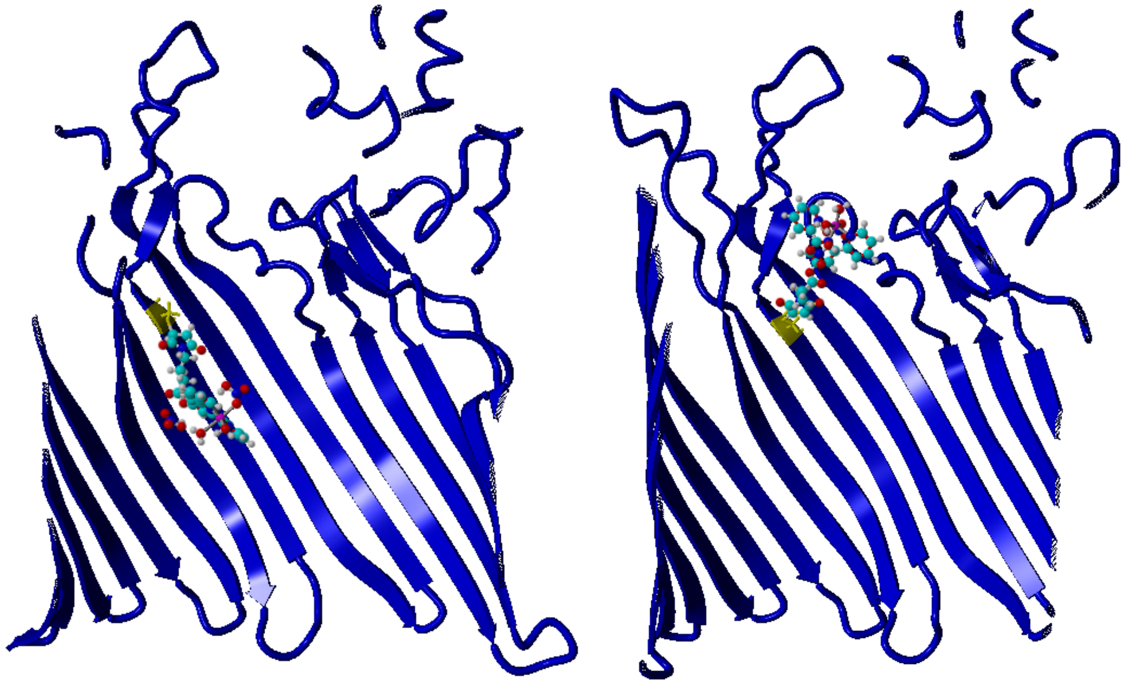

Figure S1: Illustration of the catalyst **2** within the cavity of FhuA.

NMR spectra of synthesized compounds

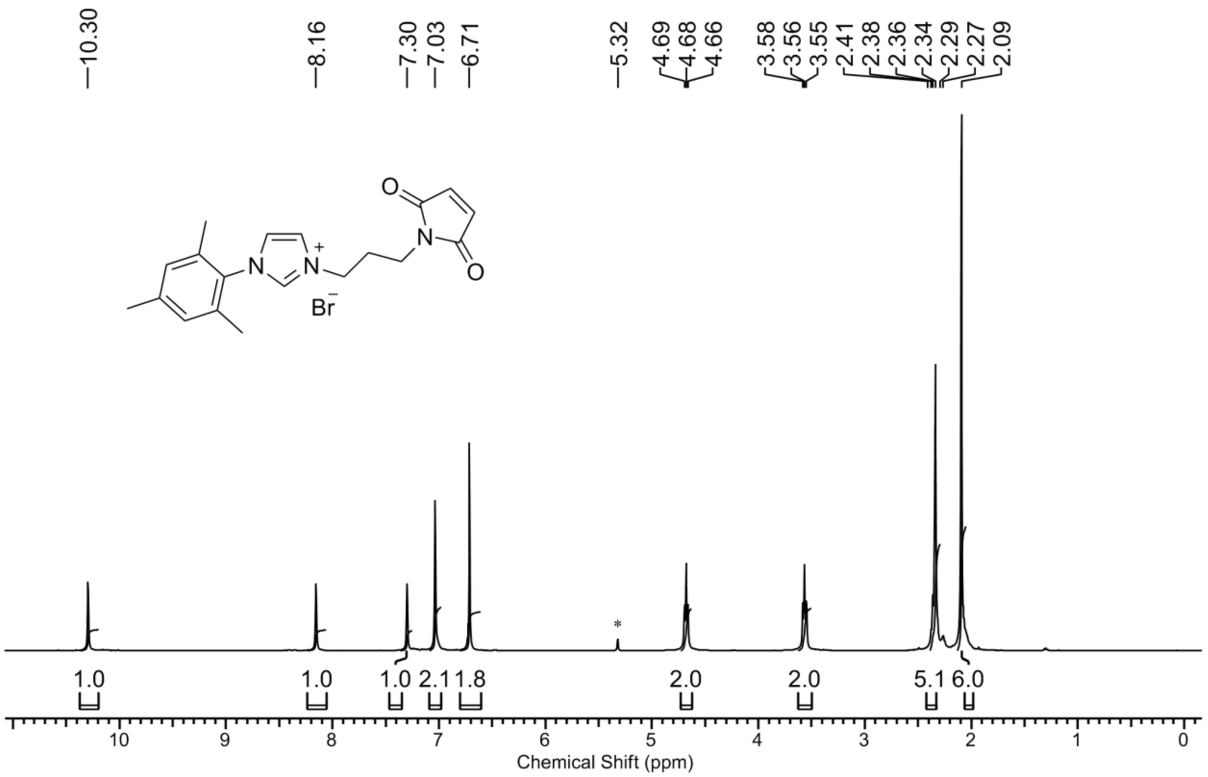

Figure S2. ^{1}H NMR spectrum (23 °C, *CD_2Cl_2) of compound **3.**

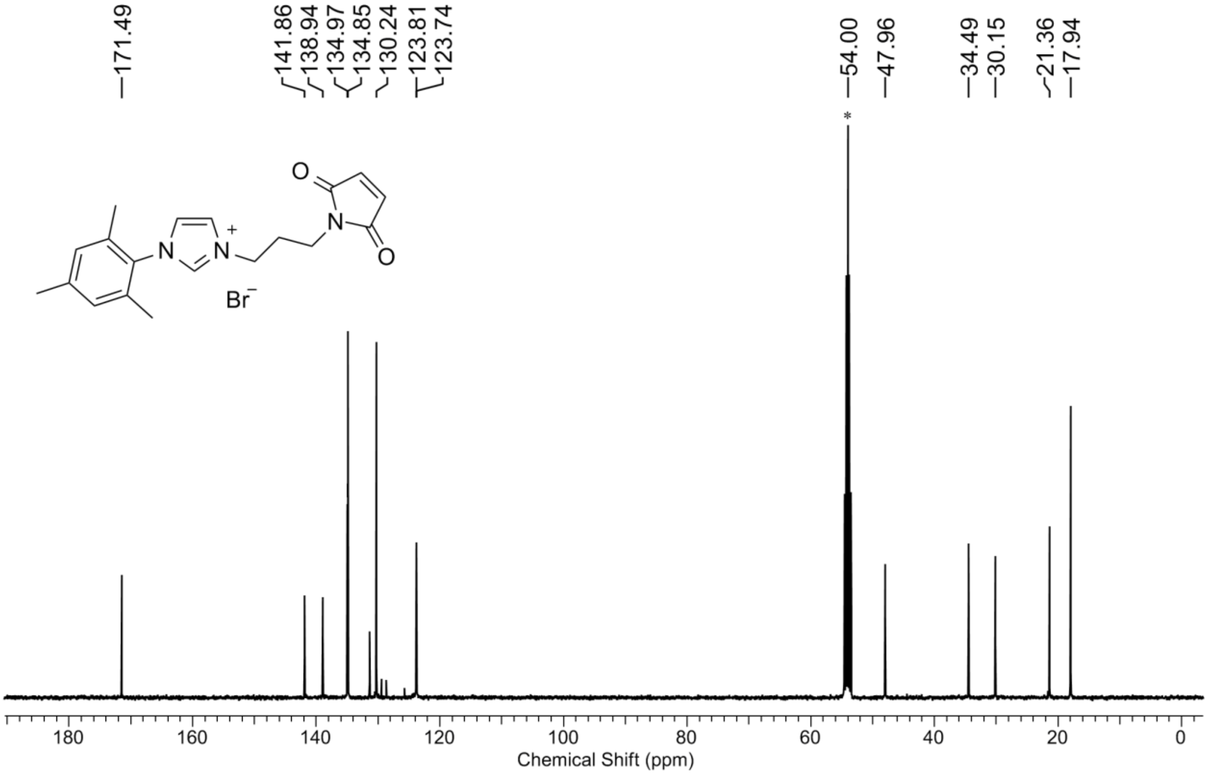

Figure S3. ^{13}C NMR spectrum (23 °C, *CD_2Cl_2) of compound **3**.

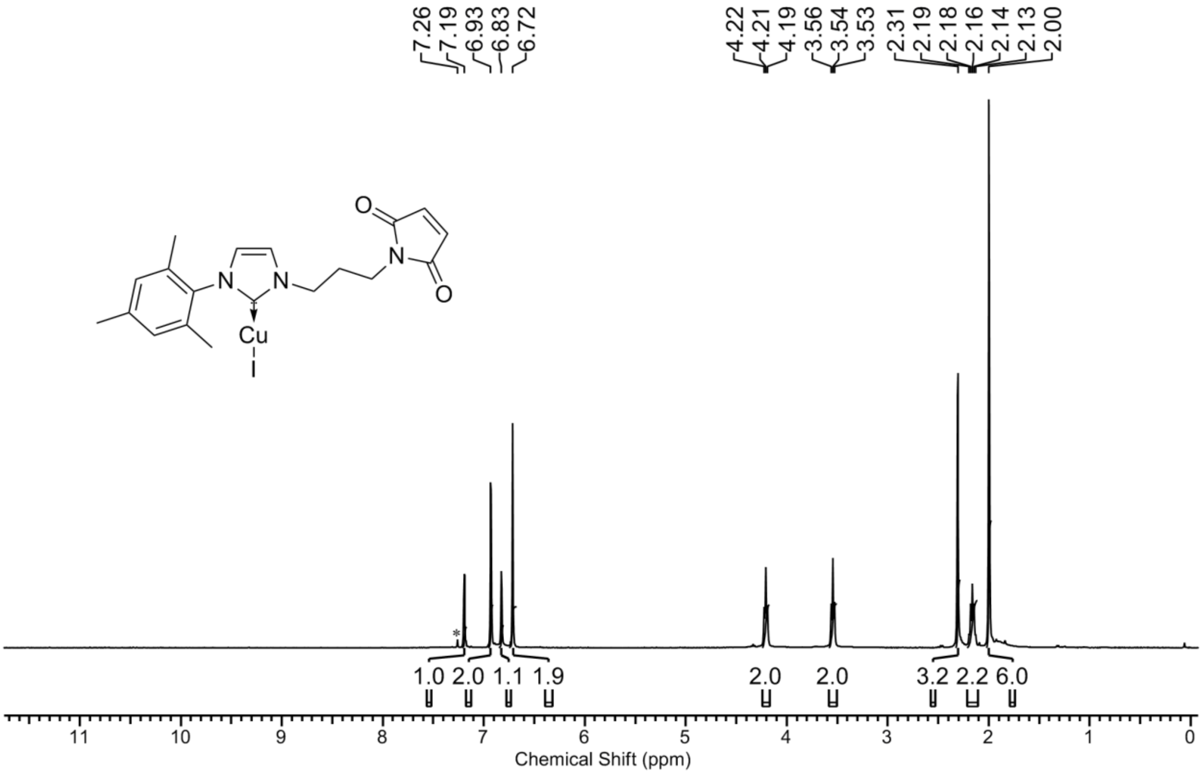

Figure S4. ^{1}H NMR spectrum (23 °C, *$CDCl_3$) of compound **4**.

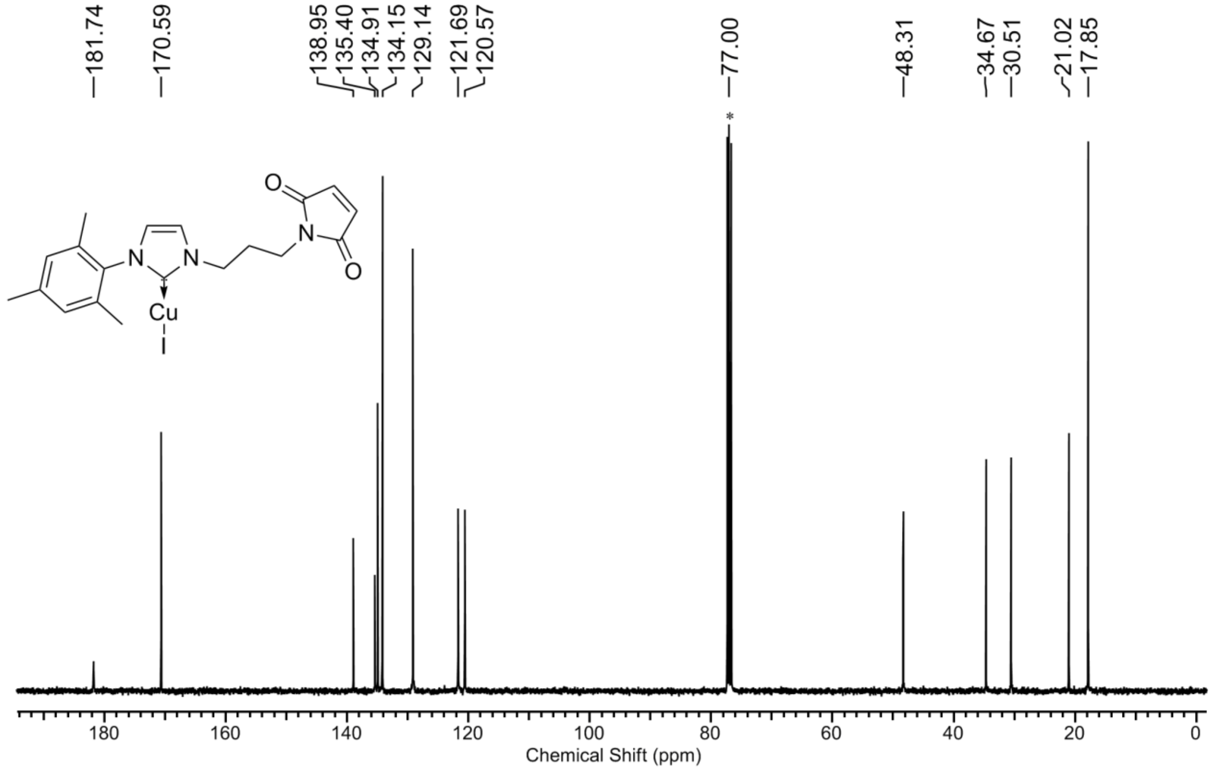

Figure S5. ^{13}C NMR spectrum (23 °C, *$CDCl_3$) of compound **4**.

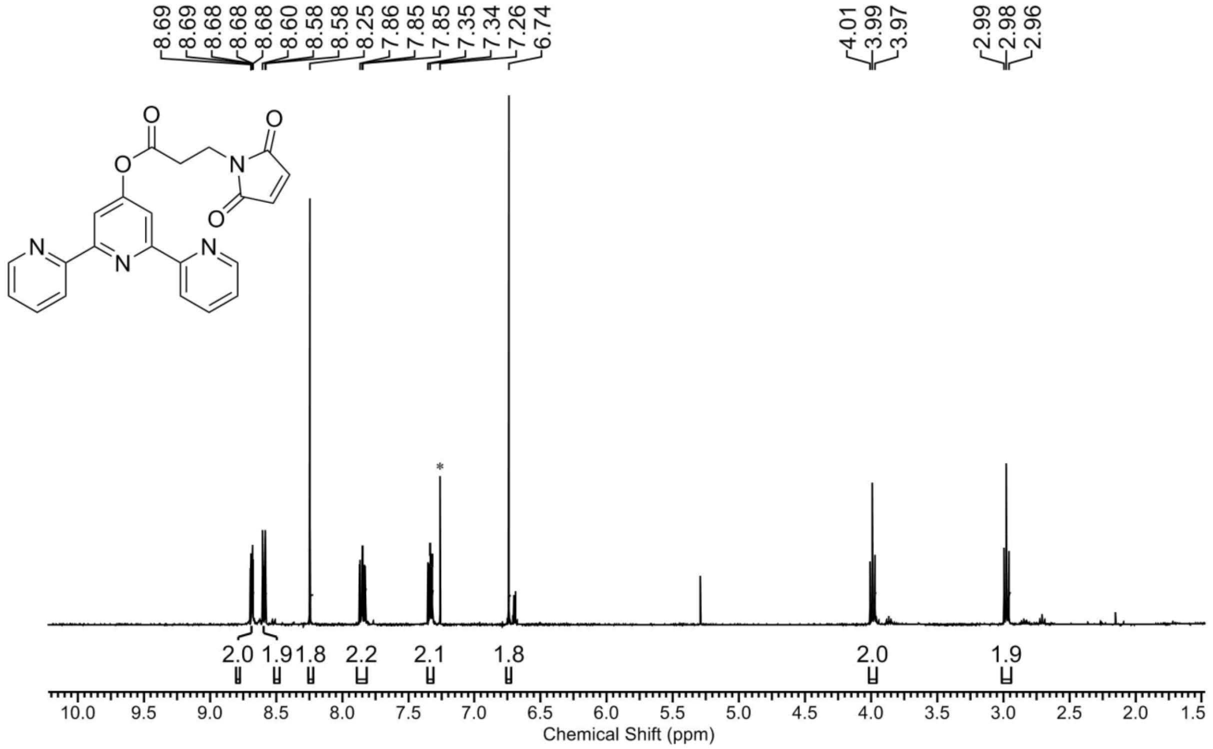

Figure S6. ^{1}H NMR spectrum (23 °C, *$CDCl_3$) of compound **8**.

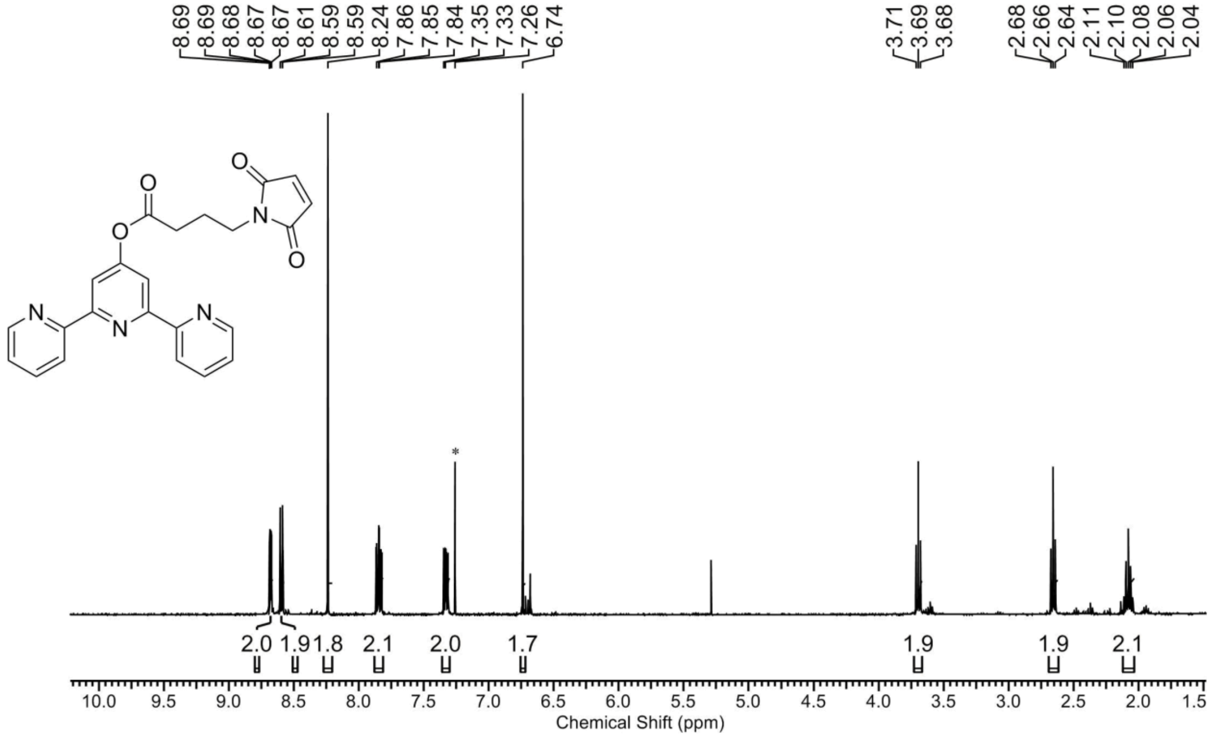

Figure S7. ^{1}H NMR spectrum (23 °C, *$CDCl_3$) of compound **9**.
